# Ringworm Infection from Animals

**Published:** 1901-02-23

**Authors:** 


### RINGWORM INFECTION FROM ANIMALS.

IT has long been thought that ringworm is often derived from animals. But it is difficult to prove that such has been the case in any given instance, although, as is well known, cultures from human ringworm can be inoculated into animals, and will in them produce characteristic lesions in which the fungus can be demonstrated. The frequency with which ringworm occurs in children renders it very difficult to eliminate other possibilities even when the chance of animal contagion is evident. Dr. Bunch[1] has, however, advanced the matter considerably by showing in a series of cases, the ætiology of which he has carefully investigated, the great similarity which has existed between the cultures obtained from the ringworm of the patient and that obtained from the animal ringworm from which the patient has been imagined to have caught the disease. The researches of Sabouraud have shown us sufficiently clearly that ringworm is not always due to the same fungus, and that these fungi exhibit very marked differences, not merely in their microscopical features and in their modes of growth in and upon the hair, but in their cultural characters. Hence Dr. Bunch's investigations tend very strongly to confirm the hypothesis that human ringworm is in many cases derived from the same disease in animals, for he shows, in regard to the cases investigated, how markedly the cultures obtained from the ringworm of the patient and from the ringworm of the animal from which the patient was presumed to have been infected resembled each other, in some cases taking one form and in others another, but always agreeing with that taken by the cultures derived from the incriminated animals. The photographs accompanying the paper referred to are very striking.

[1] Brit. Med. Journ., Feb. 9.

